# The requirement of an early detection of vulnerability base patterns in childhood to reduce relapse tendency in psychiatric pathology

**DOI:** 10.1192/j.eurpsy.2021.623

**Published:** 2021-08-13

**Authors:** L. Carpio Garcia, C. Martín Villarroel, G. Belmonte García, J. Dominguez Cutanda, M. Sánchez Revuelta, J. Matsuura, E. García

**Affiliations:** Psychiatry, COMPLEJO HOSPITALARIO DE TOLEDO, Toledo, Spain

**Keywords:** relapse tendency, Childhood psychiatry, adolescent

## Abstract

**Introduction:**

In order to understand etiopathogeny of any mental illness, it is important to be aware of the sequential emergence of symptoms, having presentations, that appear before, after or simultaneously. We could understand mental pathology as the sumatory of different factors and vulnerable cerebral substrates. Adverse external factors influence over them, causing relapses, that would lead to the evolution of diagnosis through time. However,patients usually come when pathology is already developed. Interventions are delayed, what is insufficient to modify the course of the illness.

**Objectives:**

Proving that replacing classic clinical evaluation by an open access/multiintervention model, would determine a better prevention and reduction of relapse tendency.

**Methods:**

We have arranged a prospective descriptive study of 124 users along 2 years. The idea was to test a first sample which let us check the viability of our project. We adopted a qualitative approach, linking practice and research, which have implied to perform a structured clinical process based on a dynamic reevaluation performed for different professionals in various stages using Rodman’s model.

**Results:**

MultiIntervention model reduces the prognosis factor of delayed treatment thanks to reaching a high risk group in the early stages. That model allows us to determine the way each factor relates to each other, what facilitates multiple-intervention that tries to eliminate the symptom and also the relapse.

**Conclusions:**

Late adolescence and early adulthood are stages in which many mental disorders start, however treatment delays some years. Rothman’s model may be a useful tool, what means a multiintervention treatment that mixes biological and psychosocial interventions.

